# The Paradoxical Effect of PARP Inhibitor BGP-15 on Irinotecan-Induced Cachexia and Skeletal Muscle Dysfunction

**DOI:** 10.3390/cancers12123810

**Published:** 2020-12-17

**Authors:** Dean G. Campelj, Cara A. Timpani, Aaron C. Petersen, Alan Hayes, Craig A. Goodman, Emma Rybalka

**Affiliations:** 1 Institute for Health and Sport (IHeS), Victoria University, Melbourne, VIC 8001, Australia; dean.campelj@live.vu.edu.au (D.G.C.); cara.timpani@vu.edu.au (C.A.T.); aaron.petersen@vu.edu.au (A.C.P.); alan.hayes@vu.edu.au (A.H.); 2 Australian Institute for Musculoskeletal Science, Victoria University, St Albans, VIC 3021, Australia; 3 Department of Medicine–Western Health, Melbourne Medical School, The University of Melbourne, Melbourne, VIC 3021, Australia; 4 Centre for Muscle Research (CMR), Department of Physiology, The University of Melbourne, Parkville, VIC 3010, Australia

**Keywords:** anti-cancer treatments, chemotherapy, PARP-1 inhibitor, BGP-15, skeletal muscle, cachexia, muscle wasting, myopathy, dystrophin, mechanotransduction

## Abstract

**Simple Summary:**

Both cancer and the chemotherapy used to treat it are drivers of cachexia, a life-threatening body-wasting condition which complicates cancer treatment. Poly-(ADP-ribose) polymerase (PARP) inhibitors are currently being investigated as a treatment against cancer. Here, we present paradoxical evidence that they might also be useful for mitigating the skeletal muscle specific side-effects of anti-cancer chemotherapy or exacerbate them. BGP-15 is a small molecule PARP inhibitor which protected against irinotecan (IRI)-induced cachexia and loss of skeletal muscle mass and dysfunction in our study. However, peculiarly, BGP-15 adjuvant therapy reduced protein synthesis rates and the expression of key cytoskeletal proteins associated with the dystrophin-associated protein complex and increased matrix metalloproteinase activity, while it increased the propensity for fast-twitch muscles to tear during fatiguing contraction. Our data suggest that both IRI and BGP-15 cause structural remodeling involving proteins associated with the contractile apparatus, cytoskeleton and/or the extracellular matrix which may be only transient and ultimately beneficial or may paradoxically onset a muscular dystrophy phenotype and be detrimental if more permanent.

**Abstract:**

Chemotherapy-induced muscle wasting and dysfunction is a contributing factor to cachexia alongside cancer and increases the risk of morbidity and mortality. Here, we investigate the effects of the chemotherapeutic agent irinotecan (IRI) on skeletal muscle mass and function and whether BGP-15 (a poly-(ADP-ribose) polymerase-1 (PARP-1) inhibitor and heat shock protein co-inducer) adjuvant therapy could protect against IRI-induced skeletal myopathy. Healthy 6-week-old male Balb/C mice (*n* = 24; 8/group) were treated with six intraperitoneal injections of either vehicle, IRI (30 mg/kg) or BGP-15 adjuvant therapy (IRI+BGP; 15 mg/kg) over two weeks. IRI reduced lean and tibialis anterior mass, which were attenuated by IRI+BGP treatment. Remarkably, IRI reduced muscle protein synthesis, while IRI+BGP reduced protein synthesis further. These changes occurred in the absence of a change in crude markers of mammalian/mechanistic target of rapamycin (mTOR) Complex 1 (mTORC1) signaling and protein degradation. Interestingly, the cytoskeletal protein dystrophin was reduced in both IRI- and IRI+BGP-treated mice, while IRI+BGP treatment also decreased β-dystroglycan, suggesting significant remodeling of the cytoskeleton. IRI reduced absolute force production of the soleus and extensor digitorum longus (EDL) muscles, while IRI+BGP rescued absolute force production of the soleus and strongly trended to rescue force output of the EDL (*p* = 0.06), which was associated with improvements in mass. During the fatiguing stimulation, IRI+BGP-treated EDL muscles were somewhat susceptible to rupture at the musculotendinous junction, likely due to BGP-15’s capacity to maintain the rate of force development within a weakened environment characterized by significant structural remodeling. Our paradoxical data highlight that BGP-15 has some therapeutic advantage by attenuating IRI-induced skeletal myopathy; however, its effects on the remodeling of the cytoskeleton and extracellular matrix, which appear to make fast-twitch muscles more prone to tearing during contraction, could suggest the induction of muscular dystrophy and, thus, require further characterization.

## 1. Introduction

Cachexia is a chronic and often fatal consequence of cancer and the chemotherapeutic agents used to treat it. It is a complex wasting syndrome defined as a >5% loss of body mass, which features lean tissue wasting with or without a loss of fat mass [1], as well as prominent skeletal myopathy that manifests as both wasting and dysfunction [2,3,4]. Historically, cancer-related cachexia was considered a tumor-induced phenomenon; however, mounting evidence suggests that chemotherapy drugs plays a prominent role in the progression of cachexia independent of the tumor [5,6,7,8]. Several chemotherapeutic agents from different drug classes (and with different mechanisms of action) elicit skeletal muscle toxicity [9], which, at the molecular level, involves disruption of proteostasis in favor of protein degradation and mitochondrial dysfunction through which oxidative stress/damage is escalated [10,11,12]. Despite these data, most chemotherapeutic agents in clinical utility have not been evaluated for their potential to induce skeletal myopathy. As such, muscle wasting as a side-effect of anti-cancer chemotherapy treatment is a largely overlooked factor, despite contributing to dose-limiting toxicities and poorer survival outcomes [13].

Irinotecan (IRI) is a chemotherapeutic agent commonly utilized as part of anti-cancer treatment regimens against colorectal, pancreatic and small-cell lung cancer [14,15,16]. As a potent topoisomerase I inhibitor [17], IRI treatment causes severe gastrointestinal toxicity, neutropenia and asthenia [18]. Recently, skeletal muscle wasting has also emerged as a major off-target event and is a key prognostic indicator of mortality in IRI-treated cancer patients [19,20]. Recent studies have established that the FOLFIRI (Folinic acid.leucovorin, fluorouracil and IRI regimen), in which IRI is a staple (in addition to toleucovorin and 5-fluorouracil), induces skeletal muscle wasting and dysfunction in otherwise healthy mice [11]. Although it has never been evaluated independently, IRI likely drives this myopathy, since treatment with the FOLFOX (Folinic acid/leucovorin), fluorouracil and oxaliplatin) regimen, which includes leucovorin and 5-fluorouracil but substitutes IRI for oxaliplatin, had no effect on skeletal muscle [11]. Multi-omics analysis of FOLFIRI-treated mouse muscles [21,22] and in vitro cell culture studies with IRI [23] implicate mitochondrial dysfunction, characterized by the downregulation of proteins associated with mitochondrial dynamics and oxidative phosphorylation and reduced adenosine triphosphate (ATP) synthesis, glucose metabolism and mitochondrial viability in this myopathy. However, the molecular events occurring up- and/or downstream of the mitochondria that result in muscle wasting and dysfunction are currently unknown. Therefore, in this study, our first aim was to characterize the effect of IRI treatment on the skeletal muscular system in vivo and investigate the underlying molecular mechanisms.

Currently, there is no approved treatment against cachexia, despite many adjuvant therapeutics having been clinically evaluated (reviewed recently in [24]) Targeting the mitochondria could be therapeutically advantageous in this regard since they appear implicit in IRI-induced skeletal myopathy. One potential therapeutic candidate is the small molecule BGP-15, a hydroximic acid derivative nicotinic acid-amidoxime [25], which has demonstrated therapeutic potential in treating skeletal myopathy associated with Duchenne Muscular Dystrophy [26,27], ventilation-induced diaphragm dysfunction [28,29,30,31], sarcopenia [32], diabetes [33,34,35] and oxaliplatin treatment [36]. BGP-15 inhibits poly-(ADP-ribose) polymerase-1 (PARP-1) [37], a repressor of mitochondrial function [38,39], and co-induces heat shock protein-70 (HSP-70) [40,41], which increases mitochondrial content, function and oxidative capacity [42]. HSP-70 activation also plays a role in the maintenance of skeletal muscle mass, in part, by regulating proteostasis [43]. Key to the success of any adjuvant administered to protect against the side-effects of chemotherapy is that tumor growth is not accentuated—in a pre-clinical cancer model, BGP-15 did not affect tumor growth [44], nor did it impact the anti-cancer efficacy of cisplatin [37]. Collectively, these data highlight that BGP-15 may be useful as an adjuvant candidate during anti-cancer chemotherapy to mitigate cachectic skeletal myopathy. Thus, our secondary aim was to evaluate the protective efficacy of BGP-15 adjuvant therapy against IRI-induced skeletal myopathy and investigate the underlying mechanisms through which BGP-15 functions in the chemotoxic environment.

In this study, we conclude that anti-cancer IRI treatment causes significant cachexia featuring muscle atrophy and dysfunction in mice, which could be attenuated with BGP-15 adjuvant therapy. For the first time, we show that IRI causes cytoskeletal changes involving the loss of dystrophin protein expression which negatively impact skeletal muscle mass and function. BGP-15 adjuvant therapy causes further remodeling as well as pro-mitochondrial activity but makes fast-twitch muscles more prone to tearing during fatiguing contraction. It is difficult to ascertain from our data whether BGP-15 adjuvant therapy is beneficial in the long term or whether it induces a type of muscular dystrophy and is therefore deleterious. The potential negative impact of BGP-15 on the skeletal muscular system when teamed with chemotherapeutic agents should be considered in the broader context of using PARP inhibitors as staples in anti-cancer therapy.

## 2. Results

### 2.1. Irinotecan (IRI) Induces Cachexia Which Is Mitigated by BGP-15

**Figure 1:**
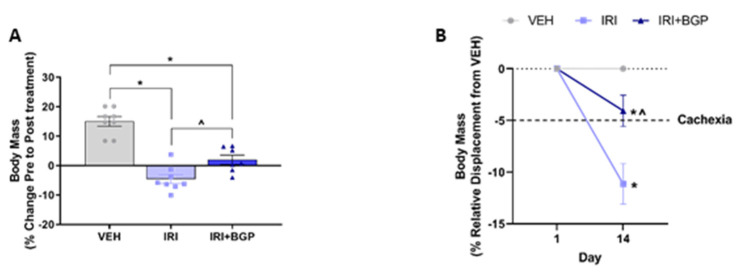

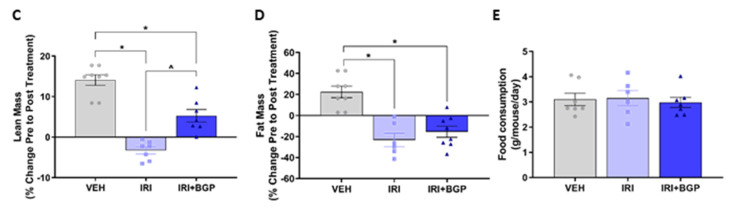
The effect of irinotecan (IRI) and IRI with BGP-15 (IRI+BGP) treatment on body composition indices and food consumption. Body composition parameters were measured pre- and post-treatment with body mass presented as (**A**) percentage change from Pre to Post treatment and (**B**) relative displacement percentage compared to vehicle (VEH) from Pre to Post treatment. (**C**) Lean and (**D**) fat mass are presented as a percentage change from Pre to Post treatment. (**E**) Food consumption was monitored throughout the treatment period. * = *p* < 0.05 compared to VEH; ^ = *p* < 0.05 compared to IRI; *n* = 7–8.

In order to determine the capacity for IRI and IRI+BGP treatment to induce and mitigate cachexia, respectively, body mass and composition, as well as skeletal muscle mass indices, were assessed. IRI-treated mice lost ~5% of their body mass over the course of the treatment period, which was partially mitigated by IRI+BGP treatment (*p* < 0.05; Figure 1A). When expressed relative to the vehicle (VEH) group, which gained ~5% of their initial body mass over the treatment period, IRI treatment caused marked cachexia with a ~10% displacement from normal/expected body mass (*p* < 0.05; Figure 1B). Combined IRI+BGP treatment attenuated body mass displacement to <5% (pre-cachexia) (*p* < 0.05 compared to IRI; Figure 1B). Unsurprisingly, lean mass data mirrored body mass data, with IRI treatment inducing a ~5% loss of lean mass compared to VEH, which was also partially mitigated by IRI+BGP treatment but still significantly lower than VEH control lean mass (*p* < 0.05; Figure 1C). There was, however, no protective effect from IRI+BGP treatment on fat mass, with both IRI and IRI+BGP treatment inducing a ~20% reduction in fat mass compared to VEH (*p* < 0.05; Figure 1D). These reductions in body composition indices were not due to differences found in food consumption between treatment groups (*p* > 0.05; Figure 1E).

### 2.2. IRI Treatment Causes Muscular Atrophy Which Is Normalized by BGP-15

**Figure 2:**
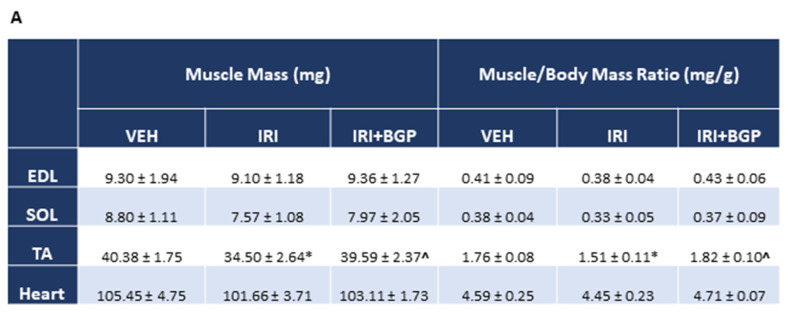

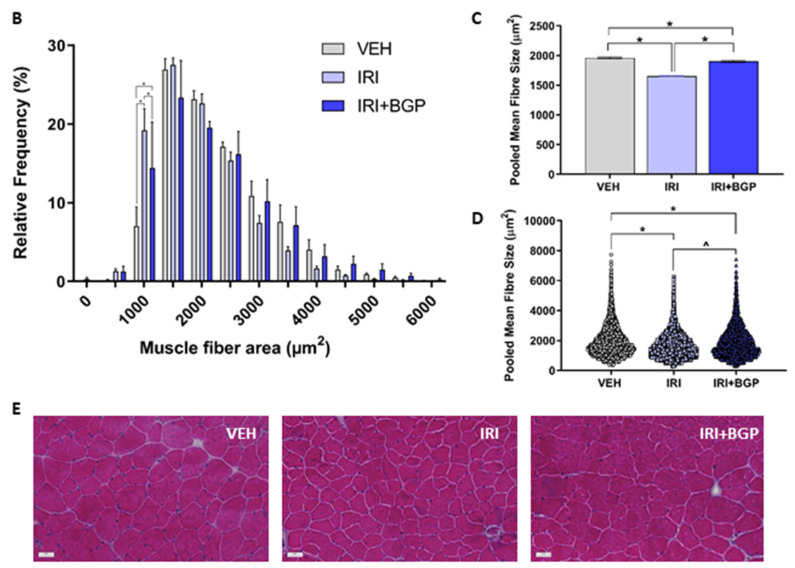
The effect of irinotecan (IRI) and IRI with BGP-15 (IRI+BGP) treatment on muscle mass and fiber size. (**A**) Skeletal muscle masses of extensor digitorum longus (EDL), soleus (SOL), tibialis anterior (TA) and heart were measured post-mortem and are presented as both raw mass and as a muscle mass to body mass ratio. TA cross-sections were hematoxylin and eosin (H&E)-stained to assess the skeletal muscle histological fiber size with data presented as (**B**) muscle fiber cross-sectional area (CSA) percentage relative frequency distribution, (**C**) the mean group pooled muscle fiber CSA (of all fibers counted) and (**D**) the group pooled fiber size distribution. (**E**) Representative images of H&E-stained TA cross-sections show no evidence of muscle damage (e.g., centronucleated fibers and immune cell infiltrate). Scale bar = 50 µm; * = *p* < 0.05 compared to VEH, ^ = *p* < 0.05 compared to IRI; *n* = 6–8 for muscle weights; *n* = 4–6 for histology.

To explore whether the IRI-induced loss of lean mass was emulated in skeletal muscle, we sought to determine whether IRI and IRI+BGP treatment could influence crude mass of a selection of hindlimb muscles or the cross-sectional area (CSA) of tibialis anterior (TA) fibers. There were no significant differences in raw mass or the muscle to body mass ratios between treatment groups for extensor digitorum longus (EDL), soleus (SOL) or heart muscles (*p* > 0.05; Figure 2A). However, both TA raw mass and the TA to body mass ratio were significantly reduced following IRI treatment (*p* < 0.05), which was protected against by IRI+BGP treatment (*p* < 0.05; Figure 2A). Histological fiber size profiling demonstrated a left shift in the CSA frequency distribution induced by IRI treatment, which was normalized to VEH control levels by IRI+BGP treatment (Figure 2B). Furthermore, mean fiber CSA and the pooled fiber size distribution were also significantly reduced following IRI treatment compared to VEH (*p* < 0.05; Figure 2C–E) and IRI+BGP treatment partially rescued both of these measures (*p* < 0.05; Figure 2C–E). There was no evidence of muscle damage elicited by IRI treatment by way of centronucleated fibers and immune cell infiltrate (Figure 2E).

### 2.3. IRI Reduces Protein Synthesis which Is Exacerbated by BGP-15

**Figure 3:**
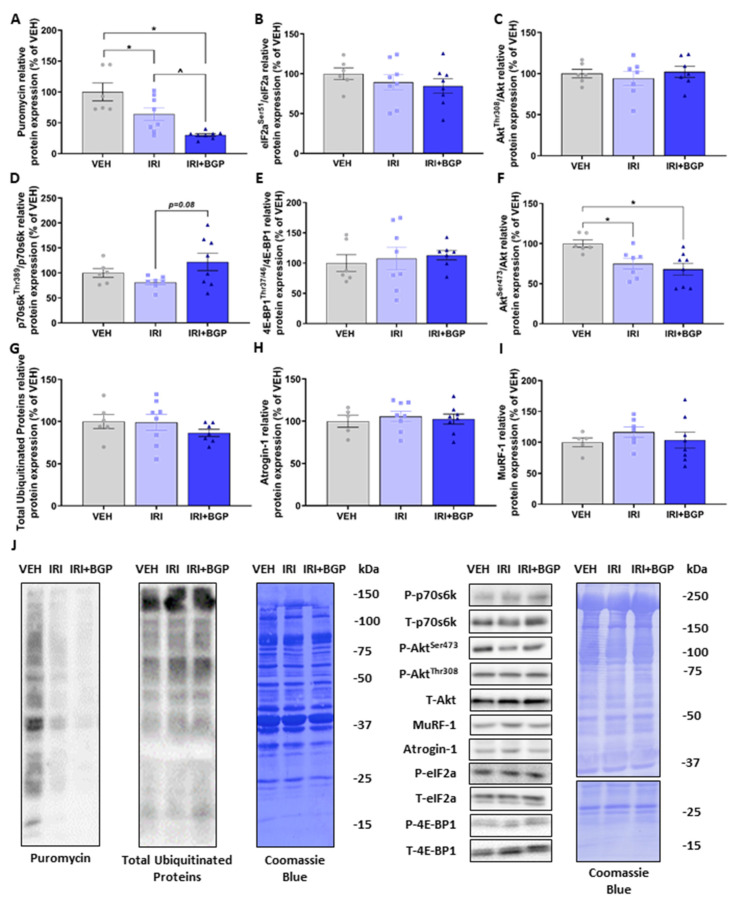
The effect of irinotecan (IRI) and IRI with BGP-15 (IRI+BGP) treatment on protein synthesis and degradation signaling. Western blotting experiments were undertaken on tibialis anterior (TA) homogenate—data are expressed as a relative percentage of the vehicle (VEH) control group. Samples were probed for (**A**) puromycin as a marker of protein synthesis via the surface sensing of translation (SUnSET) method, (**B**) eIF2a^Ser51^ relative to total eIF2a, (**C**) Akt^Thr308^ relative to total Akt, (**D**) p70s6k^Thr389^ relative to total p70s6k, (**E**) 4E-BP1^Thr37/46^ relative to total 4E-BP1, (**F**) Akt^Ser473^ relative to total Akt, (**G**) Ubiquitin, (H) Atrogin-1 and (**I**) MuRF-1. (**J**) Western blot representative images are displayed alongside a Coomassie Blue representative image, which was used as the protein loading control. * = *p* < 0.05 compared to VEH, ^ = *p* < 0.05 compared to IRI; *n* = 5–8.

To investigate changes to molecular signaling of protein turnover as a potential mechanism for IRI-induced muscle fiber atrophy, we initially assessed skeletal muscle protein synthesis signaling via the in vivo surface sensing of translation (SUnSET) method [45], which measures the rate of puromycin-labelled peptide production as an indicator of overall protein synthesis rates. IRI treatment reduced protein synthesis compared to VEH, and surprisingly, IRI+BGP treatment further reduced protein synthesis (*p* < 0.05; Figure 3A,J). These decreases in protein synthesis were independent of a change in the phosphorylation of eIF2a, a highly characterized inhibitor of protein synthesis [46] (*p* > 0.05; Figure 3B,J). Given its prominent role regulating skeletal muscle protein synthesis [47], we next probed for markers involved in the activation of mammalian/mechanistic target of rapamycin (mTOR) Complex 1 (mTORC1) signaling and mTORC1’s downstream targets. Interestingly, we demonstrated no significant differences between treatment groups in Akt^Thr308^, p70s6k1^Thr389^ or 4E-BP1^Thr37/46^ phosphorylation (*p* > 0.05; Figure 3C–E,J), although there was a trend for IRI+BGP treatment to increase p70s6k1 signaling relative to IRI (*p* = 0.08; Figure 3D,J). There was, however, a reduction in Akt^Ser473^ phosphorylation with both IRI and IRI+BGP treatments (*p* < 0.05; Figure 3F,J), a site targeted by mTORC2 (mTOR Complex 2) signaling [48]. On the alternative side of protein turnover, we saw no significant effect between treatment groups in crude static molecular markers of protein degradation, i.e., total ubiquitinated proteins, Atrogin-1 or muscle RING-finger protein-1 (MuRF-1) (*p* > 0.05; Figure 3G–J). Full-length Western blots, densitometry data and total protein data are provided in Figures S1, S2 and S4 and Table S1.

### 2.4. Assessment of Molecular Markers of Sarcolemmal Membrane Integrity

**Figure 4:**
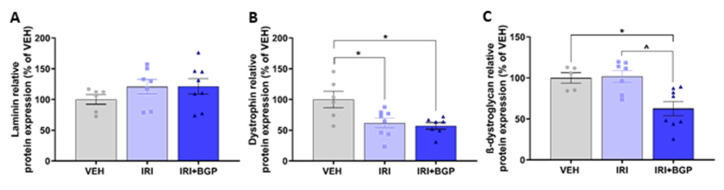

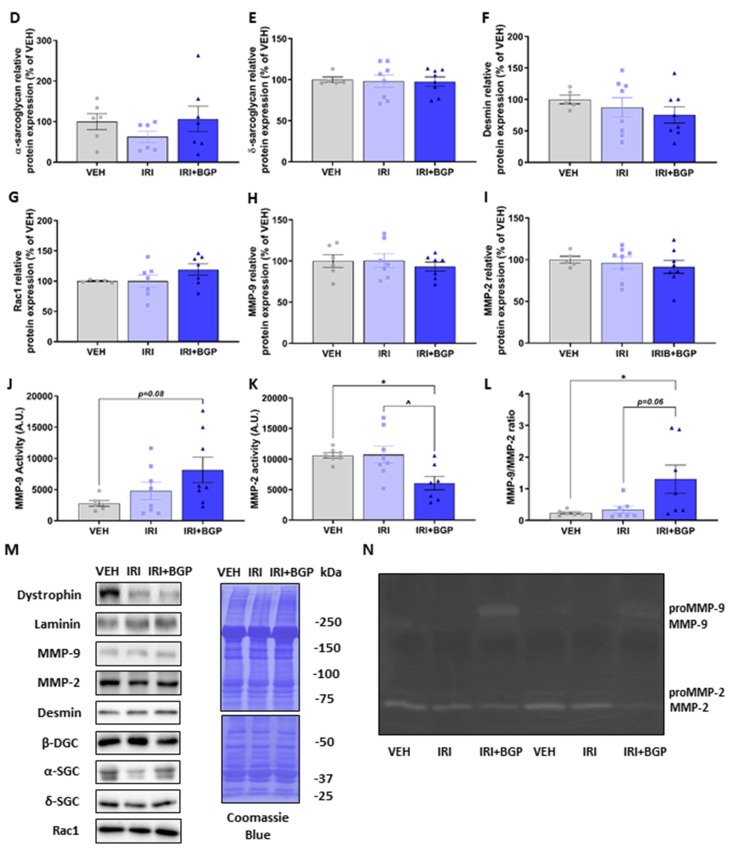
The effect of irinotecan (IRI) and IRI with BGP-15 (IRI+BGP) treatments on molecular markers of sarcolemmal integrity and extracellular matrix (ECM) remodeling. Western blotting experiments were undertaken in tibialis anterior (TA) muscle, with samples probed for (**A**) Laminin, (**B**) Dystrophin, (**C**) β-dystroglycan (β-DGC), (**D**) α-sarcoglycan (α-SGC), (**E**) δ-sarcoglycan (δ-SGC), (**F**) Desmin, (**G**) Rac1 and matrix metalloproteinase (MMP) isoforms (**H**) MMP-9 and (**I**) MMP-2. Gelatin zymography experiments were conducted using extensor digitorum longus (EDL) muscle homogenate (same anterior hindlimb muscle compartment as the TA) to assess the activity of (**J**) MMP-9 and (**K**) MMP-2. (**L**) The ratio of MMP-9 to MMP-2 was utilized to indicate the shift of gelatinolytic MMP activity. Western blotting data are expressed as a relative percentage of the vehicle (VEH) control group and (**M**) representative images are displayed alongside the Coomassie Blue representative image, which was used as the protein loading control. (**N**) Representative image of zymography data is displayed. * = *p* < 0.05 compared to VEH; ^ = *p* < 0.05 compared to IRI; *n* = 5–8.

Since IRI+BGP enhanced the IRI-induced reduction in protein synthesis rate without affecting crude markers of protein degradation, we wanted to investigate whether this might be a consequence of perturbed mechanotransduction through exploring skeletal muscle structural protein expression. Utilizing homogenized TA muscles which are from the same hindlimb anterior compartment and have similar fiber type distributions (i.e., predominately fast-twitch, glycolytic type II fibers) as EDL muscles, we initially probed for laminin, which links the extracellular matrix (ECM) to the sarcolemmal membrane. There was no significant effect on the protein expression of laminin between treatment groups (*p* > 0.05; Figure 4A,M); however, when we assessed the protein expression of dystrophin, a key structural protein that connects the sarcolemmal membrane to the actin cytoskeleton, IRI treatment was found to induce a significant reduction, which was not mitigated by IRI+BGP treatment (*p* < 0.05; Figure 4B,M). We then probed other key structural proteins located on the sarcolemmal membrane and demonstrated that IRI+BGP treatment significantly reduced the protein expression of β-dystroglycan relative to VEH- and IRI-treated mice (*p* < 0.05; Figure 4C,M), but there were no significant changes from treatment to the protein expression of sarcoglycan isoforms α and δ (*p* > 0.05; Figure 4D,E,M). There was no significant effect between treatment groups on the protein expression of the intermediate filament desmin (*p* > 0.05; Figure 4F,M), a key structural protein that connects the sarcomere to the subsarcolemmal cytoskeleton. Full-length Western blots, densitometry data and total protein data are provided in Figures S2 and S4 and Table S1.

It has previously been shown in non-muscle cells that BGP-15 can induce remodeling of plasma membrane lipid rafts, in part, by a Ras-related C3 botulinum toxin substrate 1 (Rac1) GTPase-dependent mechanism [49]. Furthermore, Rac1 signaling has been shown to mediate matrix metalloproteinases’ (MMPs) activity in multiple cell types [50,51,52,53], which are a group of proteolytic enzymes that can degrade key structural components of the contractile apparatus assembly and the ECM, such as troponin and collagen, respectively, to facilitate remodeling [54,55,56]. As such, we wanted to assess whether BGP-15 improved muscle mass and function through MMP-9 and MMP-2 activity-dependent structural remodeling. Consistent with our HSP-70 protein expression data, there was no significant effect of IRI or IRI+BGP treatment on Rac1 (*p* > 0.05; Figure 4G,M) or MMP-9 and MMP-2 protein expression (*p* > 0.05; Figure 4H,I,M) when probing TA homogenate. However, when we evaluated MMP-9 and MMP-2 activity in EDL homogenate (EDL muscles have a similar fiber type composition to TA muscles) via gelatin zymography, we found a strong trend for IRI+BGP treatment to increase MMP-9 activity relative to VEH (*p* = 0.08; Figure 4J,N) whilst also significantly reducing MMP-2 activity compared to VEH and IRI treatments (*p* < 0.05; Figure 4K,N). We also identified that IRI+BGP treatment shifted homeostatic MMP activity through increasing the MMP-9 to MMP-2 (MMP-9/MMP-2) activity ratio compared to VEH and IRI treatments (*p* < 0.05 and *p* = 0.06, respectively; Figure 4L,N), an event likely driven by contractile apparatus/cytoskeletal/ECM remodeling [55,56,57]. Full-length Western blots, densitometry data and total protein data are provided in Figures S2 and S4 and Table S1.

### 2.5. Assessment of Skeletal Muscle Contractile Function

**Figure 5:**
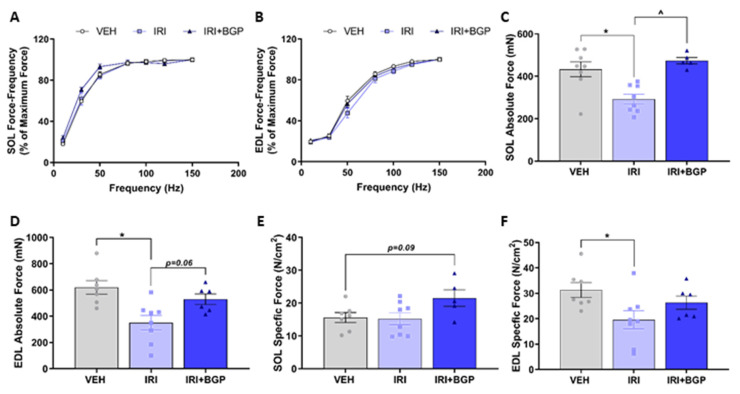

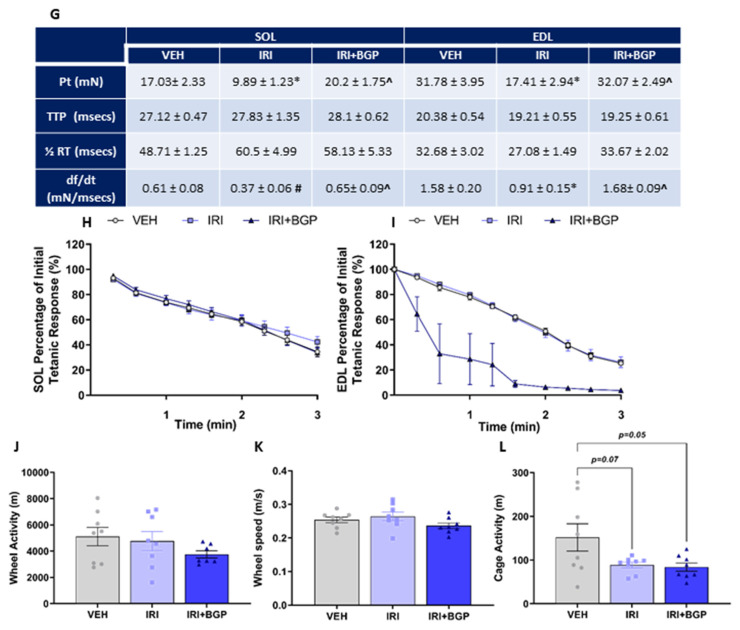
The effect of irinotecan (IRI) and IRI with BGP-15 (IRI+BGP) treatment on skeletal muscle function. Soleus (SOL) and extensor digitorum longus (EDL) muscles underwent ex vivo assessment of contractile function, with (**A**,**B**) force–frequency relationships and (**C**,**D**) absolute and (**E**,**F**) specific force production determined. (**G**) Single twitch properties, i.e., Pt = single twitch force, TTP = time to peak, ½ RT = half relaxation time and df/dt = rate of force production, were also evaluated for SOL and EDL muscles. Additionally, (**H**) SOL and (**I**) EDL muscles underwent fatigue protocols (sequential supramaximal tetanic stimulations) to assess fatigue susceptibility. To correlate ex vivo function data with overall physical activity, mice were housed in Promethion metabolic cages containing running wheels for 24 h prior to endpoint muscle collection. Voluntary wheel running (**J**) distance and (**K**) speed were assessed in addition to (**L**) cage activity over these 24 h. * = *p* < 0.05 compare to VEH; # *p* = 0.08 compared to VEH, ^ = *p* < 0.05 compared to IRI; *n* = 5–8.

We examined the contractile function of the predominately slow-twitch SOL and the predominately fast-twitch EDL muscles. We found no significant differences between treatment groups in the force–frequency relationships of either SOL or EDL muscles (*p* > 0.05; Figure 5A,B); however, we demonstrated that the absolute force production of SOL and EDL muscles was significantly reduced by IRI treatment (*p* < 0.05; Figure 5C,D). IRI+BGP treatment was protective against IRI-induced skeletal muscle dysfunction with absolute force normalization in the SOL muscle (*p* < 0.05; Figure 5C) and a strong trend toward improved absolute force production of the EDL muscle (*p* = 0.06; Figure 5D) relative to IRI-treated animals. When we accounted for the physiological CSA, there was no significant effect on specific force production of the SOL compared to VEH (*p* > 0.05; Figure 5E), but IRI treatment did reduce specific force production of the EDL (*p* < 0.05; Figure 5F). Interestingly, there was a trend for IRI+BGP treatment to improve the specific force production of the SOL relative to both IRI and VEH groups (*p* = 0.09; Figure 5E); however, IRI+BGP exhibited no ameliorative effect on the IRI-induced reduction in specific force production of the EDL (*p* > 0.05; Figure 5F). Mirroring the absolute force data, we demonstrated a reduction in the twitch force (Pt) and the rate of force development (df/dt) for EDL (*p* < 0.05; Figure 5G) and SOL (*p* < 0.05 and *p* = 0.08, respectively; Figure 5G) from IRI treatment, which was mitigated by IRI+BGP treatment, normalizing twitch force to VEH control levels (*p* < 0.05; Figure 5G). We then assessed two factors involved in calcium (Ca^2+^) handling (i.e., time to peak tension (TTP) and half relaxation time (½RT)), which may play a role in the underlying mechanism of IRI-induced dysfunction; however, there was no significant effect of IRI or IRI+BGP treatment on these measures (*p* > 0.05; Figure 5G). We also assessed fatigue susceptibility in SOL and EDL muscles by conducting a fatiguing stimulation protocol that induces a progressive reduction in force output of ~65–75% (Figure 5H,I). There was no significant effect of IRI or IRI+BGP treatment on the fatigability of SOL muscles (*p* > 0.05; Figure 5H), nor of IRI treatment on EDL muscles (*p* > 0.05; Figure 5I). However, it was observed that IRI+BGP-treated EDL muscles had an increased susceptibility to rupture at the musculotendinous junction during the fatigue protocol, with 50% (*n* = 3/6) tearing during the initial 3–4 stimulated contractions and the remaining 50% (*n* = 3/6) tearing but continuing to be partially responsive to stimuli (Figure 5I). Despite these ex vivo functional observations, there was no significant effect of IRI or IRI+BGP-15 on voluntary exercise, with wheel distance (*p* > 0.05; Figure 5J) and speed (*p* < 0.05; Figure 5K) unaffected by treatment over a 24-h assessment period. There was, however, a strong trend for IRI to reduce overall cage activity, which was not mitigated by IRI+BGP-15 treatment (*p* = 0.07 for IRI and *p* = 0.05 for IR+BGP-15 versus VEH; Figure 5L).

### 2.6. Assessment of Skeletal Muscle Metabolic Phenotype

**Figure 6:**
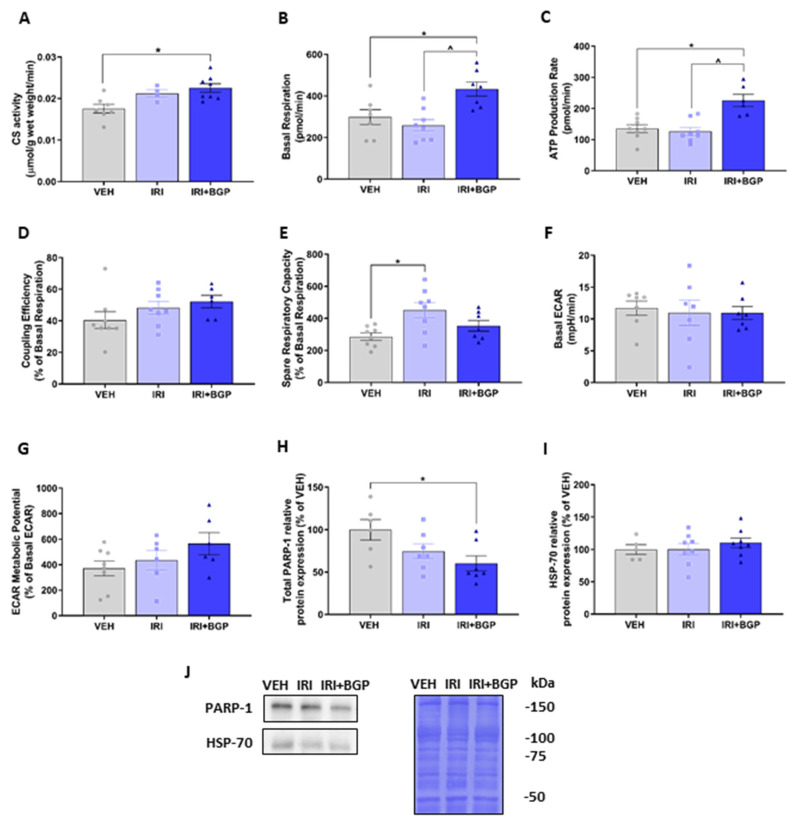
The effect of irinotecan (IRI) and IRI with BGP-15 (IRI+BGP) treatment on skeletal muscle mitochondrial metabolic phenotyping. (**A**) Tibialis anterior (TA) homogenate was analyzed for citrate synthase (CS) activity as a marker of mitochondrial density. Isolated flexor digitorum brevis (FDB) fibers were utilized for mitochondrial metabolic analyses with oxidative respiration indices, i.e., (**B**) basal respiration, (**C**) adenosine triphosphate (ATP) production rate, (**D**) coupling efficiency, (**E**) spare respiratory capacity, as well as glycolytic respiration indices, i.e., (**F**) basal extracellular acidification rate (ECAR) and (**G**) ECAR metabolic potential, measured. Furthermore, Western blotting experiments were undertaken and TA homogenate was probed for (**H**) total poly-(ADP-ribose) polymerase-1 (PARP-1) and (**I**) heat shock protein-70 (HSP-70)—these data are expressed as a relative percentage of the vehicle (VEH) control group. (**J**) Western blot representative images are displayed alongside a Coomassie Blue representative image, which was used as the protein loading control. * = *p* < 0.05 compare to VEH; ^ = *p* < 0.05 compared to IRI; *n* = 4–8 for CS activity and *n* = 5–8 for metabolic phenotyping and Western blotting.

Since we have previously demonstrated that IRI treatment can reduce skeletal muscle mitochondrial viability in vitro [58], we undertook mitochondrial profiling on muscles derived from IRI- and IRI+BGP-treated mice to determine whether similar effects could be observed. Initially, we assessed citrate synthase (CS) activity in TA homogenate as a marker of mitochondrial density. While there was no significant effect of IRI treatment, IRI+BGP treatment significantly increased CS activity compared to the VEH control group (*p* < 0.05; Figure 6A). Additionally, we conducted mitochondrial metabolic phenotyping experiments in isolated flexor digitorum brevis (FDB) muscle using extracellular flux technology to determine the relative contribution of oxidative and glycolytic metabolism. There was no effect of IRI treatment on the resting basal respiration compared to VEH (*p* > 0.05); however, IRI+BGP treatment significantly increased basal respiration compared to VEH and IRI groups (*p* < 0.05; Figure 6B). Similarly, ATP production rate was significantly increased by IRI+BGP treatment compared to the VEH and IRI groups (*p* < 0.05; Figure 6C); however, there was no significant effect of any treatment group on the coupling efficiency (*p* > 0.05; Figure 6D). Interestingly, IRI treatment increased spare respiratory capacity relative to VEH (*p* < 0.05; Figure 6E), although IRI+BGP treatment had no effect on this mitochondrial functional parameter (*p* > 0.05; Figure 6E). We also demonstrated no impact of either IRI or IRI+BGP treatment on glycolytic metabolism as there was no significant effect of treatment on basal extracellular acidification rate (ECAR) or ECAR metabolic potential, which is indicative of the maximal ECAR capacity (*p* > 0.05; Figure 6F,G, respectively).

To examine whether the mechanisms underlying the enhanced mitochondrial function from IRI+BGP treatment adhered to the previously confirmed targets of BGP-15 (i.e., HSP-70 co-induction and PARP-1 inhibition) [26,37], we next undertook Western blotting in TA homogenates. We showed that IRI+BGP treatment reduced total PARP-1 protein expression (*p* < 0.05; Figure 6H,J) but did not alter HSP-70 protein expression (*p* > 0.05; Figure 6I,J). IRI treatment alone did not significantly affect either target (*p* > 0.05; Figure 6H–J). Full-length Western blots, densitometry data and total protein data are provided in Figures S3 and S4 and Table S1.

### 2.7. Assessment of Redox Balance and Mitochondrial Content Signaling Pathways in Skeletal Muscle

**Figure 7:**
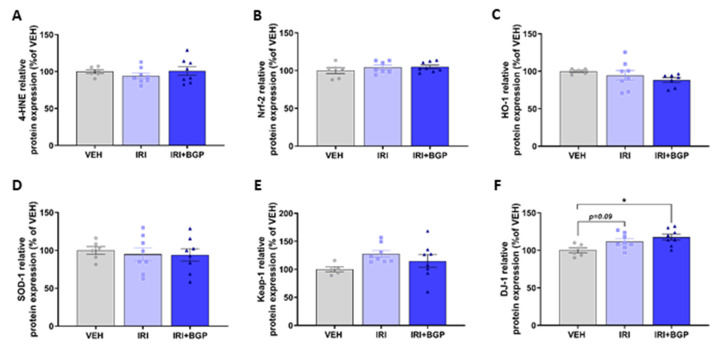

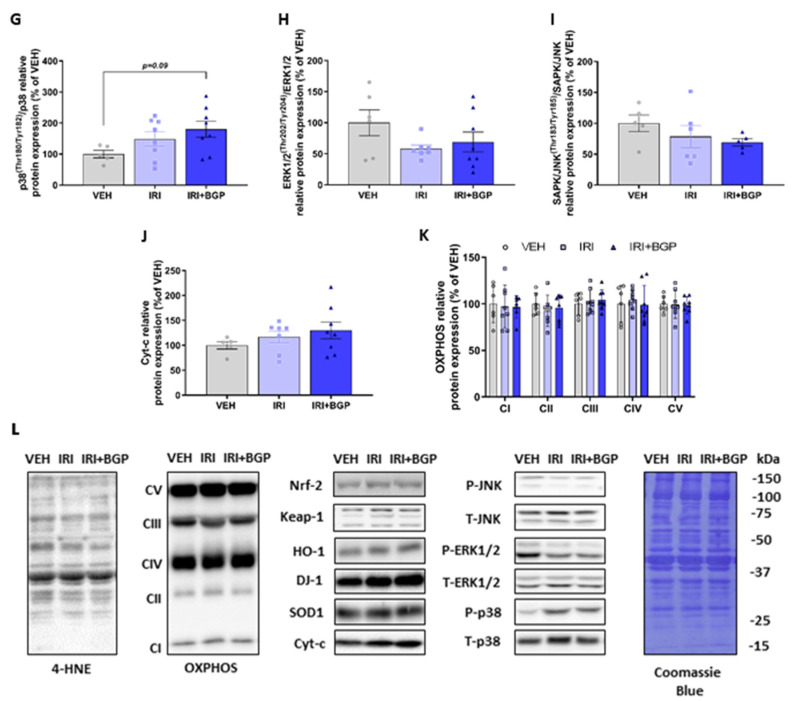
The effect of irinotecan (IRI) and IRI with BGP-15 (IRI+BGP) treatment on oxidative and mitochondrial stress signaling pathways. Western blotting experiments were undertaken and tibialis anterior (TA) homogenate was probed for oxidative stress signaling markers; (**A**) 4-Hydroxynoneal (4-HNE), (**B**) nuclear factor erythroid 2 (NFE2)-related factor 2 (Nrf-2), (**C**) hemeoxygenase-1 (HO-1), (**D**) superoxide dismutase-1 (SOD1), (**E**) Kelch-like ECH-associated protein 1 (Keap-1), (**F**) protein deglycase (DJ-1), and mitogen-activated protein kinases (MAPKs)—which are sensitive to oxidative stress—(**G**) p38 (Thr^180^/Tyr^182^) relative to total p38, (**H**) extracellular signal-regulated protein kinase (ERK1/2; Thr^202^/Tyr^204)^ relative to total ERK1/2 and (**I**) c-Jun N-terminal kinase (JNK;Thr^183^/Tyr^185^) relative to total JNK are displayed. Additionally, mitochondrial stress and content markers (**J**) Cyt-c and (**K**) the OXPHOS cocktail, i.e., Complex V (CV) subunit ATP5A, Complex IV (CIV) subunit MTCO1, Complex III (CIII) subunit UQCRC, Complex II (CII) subunit SDHB and Complex I (CI) subunit NDUFB8, were probed for. These data are expressed as a relative percentage of the vehicle (VEH) control group. (**L**) Western blot representative images are displayed alongside a Coomassie Blue representative image, which was used as the protein loading control. * = *p* < 0.05 compare to VEH; *n* = 5–8.

Since skeletal muscle dysfunction caused by chemotherapeutic agents has previously been associated with oxidative stress potentiated through increased reactive oxygen species (ROS) production [59], we wanted to investigate molecular markers of redox balance and mitochondrial content in skeletal muscle. There was no effect of IRI or IRI+BGP treatment on the protein expression of 4-Hydroxynoneal (4-HNE), a marker of lipid peroxidation, which is a key hallmark of oxidative stress [60] (*p* > 0.05; Figure 7A,L); nuclear factor erythroid 2 (NFE2)-related factor 2 (Nrf-2), the master transcriptional regulator of the anti-oxidant response to cellular stress (*p* > 0.05; Figure 7B,L); or the downstream antioxidant targets of Nrf-2, hemeoxygenase-1 (HO-1) and superoxide dismutase-1 (SOD1) (*p* > 0.05; Figure 7C,D,L). Similarly, there was no significant difference in the protein expression of the negative regulator of Nrf-2, Kelch-like ECH-associated protein 1 (Keap-1), from IRI or IRI+BGP treatment (*p* > 0.05; Figure 7E,L). Interestingly, the protein expression of DJ-1, a highly conserved protein that can positively regulate Nrf-2 activity [61], was significantly increased by IRI+BGP treatment compared to VEH (*p* < 0.05; Figure 7F,L), whilst there was a modest trend for IRI treatment to increase DJ-1 protein expression relative to VEH (*p* = 0.09; Figure 7F,L).These data were further supported by normal expression of mitogen-activated protein kinase (MAPK) signaling, i.e., p38, ERK 1/2 and JNK (*p* > 0.05; Figure 7G–I,L), which is sensitive to acute increases in oxidative stress [62], between treatment groups. However, there was a modest trend for IRI+BGP treatment to increase p38 phosphorylation relative to VEH (*p* = 0.09; Figure 7G,L). Additionally, there were no significant differences between treatment groups in the protein expression of molecular markers of mitochondrial remodeling (i.e., cytochrome c (Cyt-c); *p* > 0.05; Figure 7J,L) and content (i.e., commonly probed protein subunits of the mitochondrial Complexes I–V; *p* > 0.05; Figure 7K,L). Full-length Western blots, densitometry data and total protein data are provided in Figures S1, S3 and S4 and Table S1.

## 3. Discussion

This is the first study to examine the effect of IRI treatment and BGP-15 adjuvant therapy on skeletal muscle mass and function. Our novel findings show that BGP-15 adjuvant therapy mitigates the IRI-induced wasting phenotype, which is underscored by a protective maintenance of skeletal muscle mass and fiber size. Fascinatingly, we observed a paradoxical finding where BGP-15 adjuvant therapy exacerbated the IRI-induced reduction in protein synthesis independent of mTORC1 signaling but ameliorated the IRI-induced skeletal muscle dysfunction, whilst also potentiating oxidative metabolism, apparently through suppression of PARP-1. While we showed indications of cytoskeletal and/or ECM remodeling (through changes to the dystrophin-associated protein complex (DAPC) and the MMP-9 to MMP-2 ratio) driven by both IRI and the addition of BGP-15, it is difficult to conclude whether these changes are short-lived and ultimately beneficial or whether they detrimentally progress a muscular dystrophy-like phenotype. Indeed, IRI+BGP-15 muscles were more prone to tearing due to the shear stress associated with ex vivo fatiguing contraction, although there was no evidence of histopathology.

### 3.1. Skeletal Muscle Mass versus Protein Synthesis: The Paradoxical Effect of BGP-15 Adjuvant Therapy

We have demonstrated in this study that IRI induced a cachectic phenotype characterized by skeletal muscle wasting, i.e., diminished crude TA mass and fiber CSA, mirroring previous data where IRI administered as part of the FOLFIRI chemotherapy combination regimen contributed to the induction of cachexia [11,63]. One of the underlying mechanisms of skeletal muscle mass loss in the cachectic phenotype has been proposed to be impaired protein synthesis rates, which was first demonstrated by Emery et al. [64] in cachectic cancer patients. More recently, in the absence of cancer, chemotherapeutic agents such as doxorubicin have been shown to reduce skeletal muscle protein synthesis rates independent of key mTORC1 and eIF2a regulatory signaling [65,66]. Consistent with these data, we found that IRI-induced muscle wasting was independent of reduced protein synthesis through mTORC1 inhibition. This suggests that IRI treatment alone, or in a combination regimen, can significantly impact skeletal muscle mass, placing IRI amongst doxorubicin and cisplatin as chemotherapeutic agents with skeletal muscle toxicity profiles [9]. Surprisingly, however, we found that BGP-15 adjuvant therapy further reduced basal protein synthesis rates. One question that arises from these data is: how did BGP-15 adjuvant therapy attenuate crude muscle and fiber wasting/atrophy when protein synthesis was even lower than with IRI treatment alone? While this question remains herein unanswered, if muscle mass is broadly determined by the balance between protein synthesis and degradation rates, our data suggest that BGP-15 adjuvant therapy likely decreased the rate of protein degradation to an even larger extent than the reduction in protein synthesis, leading to a net increase in protein accretion. BGP-15 has previously been shown to co-induce HSP-70 [26,29,67], and we postulate that the activation of HSP-70 may be a key factor in regulating (i.e., lowering) protein degradation [43]. For example, the overexpression of HSP-70 has been shown to partially mitigate the hindlimb unloading-induced escalation of proteolytic activity [68]. Unfortunately, we were unable to detect the co-induction of HSP-70 by BGP-15 adjuvant therapy. In a C2C12 muscle cell culture, we have observed that HSP-70 expression is significantly elevated at 30 min following treatment with 1mM BGP-15 (administered with chemotherapeutic agent, 5-fluorouracil), reaching a peak at 1 h and subsiding by 2 h (D. Campelj [69]). Our observations are consistent with others describing BGP-15-mediated HSP-70 induction < 24 h after treatment [26,29,67]. Thus, HSP-70 induction by BGP-15 is acute. transient and, therefore, undetectable in our study as we harvested muscles 3 days post the final treatment. A recent study by Salah et al. [28] illustrated that a 10-day treatment course of daily, high-dose (40 mg/kg) BGP-15 could rescue a range of proteolytic post-translational modifications induced by ventilation-induced diaphragm dysfunction. In contrast, our data show no effect of BGP-15 adjuvant therapy on crude static markers of protein degradation (i.e., the protein expression of atrogin-1, MuRF1 and total ubiquitinated proteins) at the end of the treatment period. However, we cannot rule out decreased proteasomal and/or lysosomal activity, despite no change in the steady state levels of these markers, or that changes in the markers may have occurred at an earlier time point. Clearly, more studies are now required to explore this fascinating phenomenon further.

### 3.2. The Relationship between Protein Synthesis and Cytoskeletal Re-Modelling: A Potential Role for Mechanotransduction?

Another question that arises from our data is: what was/were the mTORC1-independent mechanism(s) responsible for the IRI- and BGP-15-mediated reduction in basal protein synthesis rates? With regards to IRI treatment, one potential answer may lie in the suppression of Akt phosphorylation at the Ser^473^ residue. Akt^Ser473^ phosphorylation is indicative of mTORC2 activity [48]; mTORC2 has recently emerged as a regulator of plasma membrane homeostasis [70], and specific to skeletal muscle, sarcolemmal integrity is considered to be sensitive to Akt^Ser473^ phosphorylation [71]. Furthermore, mTORC2 activity has recently been implicated in mechanical overload-induced increases in protein synthesis and muscle growth (for a review, see [72]). Considering these two points, we postulate that the reduction in protein synthesis induced by IRI treatment and exacerbated by BGP-15 adjuvant therapy may be reflective of compromised mechanotransduction underlined by reduced Akt^Ser473^ phosphorylation and compromised cytoskeletal stability, although the timing of the cascade of events is unknown. Indeed, we demonstrated a decrease in dystrophin expression from IRI treatment (both alone and in combination with BGP-15) alongside a decrease in β-dystroglycan from BGP-15 adjuvant therapy, imitating the step-like reduction observed in protein synthesis rates. Altered expression of cytoskeletal structural proteins in skeletal muscle of chemotherapy-treated mice has previously been demonstrated via transcriptomic [73] and proteomic [22] screening; however, follow-up analysis has not been conducted. Since it has long been speculated that skeletal muscle protein synthesis regulation is tension-dependent [74], better understanding of the underlying mechanistic control of the mechanotransduction signaling pathways that influence tension is of significant interest [72,75,76,77,78,79,80]. Importantly, our data are the first to identify the DAPC in the complex interplay between mTORC2 signaling, mechanotransduction and protein synthesis.

### 3.3. The Functional Consequence of Cytoskeletal Remodeling by IRI Treatment and BGP-15 Adjuvant Therapy

In this study, we demonstrate that IRI administration reduces skeletal muscle force production in a muscle/fiber-type-independent manner, which is consistent with the dysfunction induced from other chemotherapeutic agents [11,81,82]. A contributing factor to IRI-induced skeletal muscle dysfunction may be the reduction in dystrophin protein expression, a key member of the DAPC, which acts as an anchoring protein supporting the stability of sarcomeric and sarcolemmal proteins. Swiderski et al. [83] highlight several amino acid residues in the cysteine-rich and C-terminus domains of the dystrophin protein that are susceptible to post-translational modifications, including phosphorylation by multiple kinases, such as Ca^2+^/calmodulin-dependent kinase, which can impact skeletal muscle function [84]. Indeed, the loss of dystrophin has been associated with dysregulated Ca^2+^ signaling [85], which has also been observed as a key perturbation underlying cisplatin- and doxorubicin-induced skeletal muscle dysfunction [86,87]. Thus, it would be of interest in future investigations to determine the post-translational modifications to the dystrophin protein when challenged by multiple chemotherapeutic agents, including IRI. Since BGP-15 adjuvant therapy preserved skeletal muscle contractile function, which has previously been shown in other myopathic models [27,31,32,67], the reduction in both dystrophin and β-dystroglycan expression appears not to have the same impact on muscle function as it does in muscular dystrophy models. That being said, an interesting finding of our study was that IRI+BGP-15-treated EDL muscles had an increased susceptibility to tear from the shear stress of repeated contractions during our ex vivo fatigue protocol. This may be due, in part, to the increased force production and the combined reduction in dystrophin and β-dystroglycan expression in the IRI+BGP-15-treated muscles compared to the IRI-only-treated muscles. Indeed, protein constituents of the DAPC, such as dystrophin and β-dystroglycan, play an integral role in preserving skeletal muscle stiffness and elasticity [88], assisting in the maintenance of cytoskeletal integrity during muscle contraction [89]. In addition, we demonstrated that BGP-15 adjuvant therapy increased the MMP-9/MMP-2 activity ratio, which is proposed to play a role in remodeling of the ECM and cytoskeletal structural and contractile proteins in skeletal [56] and cardiac muscle [55]. While the order of remodeling events cannot be ascertained from our data, these events combined might account for proneness to musculotendinous rupture in IRI+BGP-15-treated muscles, Future studies are required to elucidate the muscle- and ECM-specific remodeling that is induced by IRI and BGP-15.

### 3.4. BGP-15 Adjuvant Therapy Potentiates Skeletal Muscle Oxidative Metabolism through PARP-1 Inhibition

In this study, BGP-15 adjuvant therapy enhanced skeletal muscle oxidative metabolism, highlighted by the increase in basal oxygen consumption rate, ATP production and CS activity. The pro-oxidative metabolic effect of BGP-15 adjuvant therapy appears to be driven via the inhibition of PARP-1 and perhaps even as a latent effect of the transient co-induction of HSP-70 [26,29,67] in response to IRI treatment. PARP-1 inhibition exerts cellular protection in environments with excess PARP-1 activity, which is considered to promote destabilization of the mitochondrial membranes, leading to the impairment of mitochondrial function [90]. While we saw no evidence of increased PARP-1 or reduced mitochondrial function elicited by IRI treatment, PARP-1 inhibition by BGP-15 still inferred pro-mitochondrial activity. This could be due to the timepoint at which we harvested tissue (i.e., 3 days post final IRI injection), which may have preceded the acute mitochondrial perturbations elicited by IRI, as previously published by us in C2C12 myoblasts and myotubes [58]. Furthermore, the FOLFIRI regimen, of which IRI is a constituent, has been previously shown to induce acute skeletal muscle mitochondrial perturbations, highlighted by impaired mitochondrial biogenesis and enhanced pro-oxidant signaling [11]. This suggests that the metronomic delivery of IRI potentially instigates mitohormesis in skeletal muscle, where, initially, there is an acute metabolic challenge exerted upon mitochondria from IRI administration; however, with repeat insults from IRI, mitochondria adapt and become more adept at handling the prolonged metabolic challenge [91]. In this scenario, PARP-1 inhibition would transiently enhance the mitochondrial adaptations to the metabolic challenges by protecting against nicotinamide adenine dinucleotide (NAD) depletion and, through the promotion of DJ-1, aiding metabolic re-programming [92]. This hypothesis is based on the assumption that these mitohormetic protective adaptations include increasing PARP activity, for example, PARylation, which is activated during mitochondrial DNA repair and integrity maintenance [93] at the expense of depleting NAD and impairing mitochondrial function.

### 3.5. Limitations

There were some limitations to our research that are worthy of mention. In the first instance, we conducted our study using 6-week-old mice and treated them over 2 weeks, up to 8 weeks of age. Mice are considered sexually mature at 8 weeks of age; hence, we treated our mice during the pubertal developmental period but measured the impact of treatment at sexual maturity. Cachexia is well documented in pediatric cancer patients and involves both tumor- and chemotherapy-mediated pathogenesis [94]. Notably, however, pediatric cancer cachexia differs from adult cachexia in that it predominantly manifests as stunted growth [95] which, along with significant skeletal muscle dysfunction, can persist throughout the lifespan [96]. While the cachexia observed in our IRI-treated mice may have included an element of growth repression, we also observed loss of body, lean and fat mass from the pre- to post-treatment period, which is consistent with the accepted definition of cachexia [1]. Secondly, our experimental design lacked a BGP-15-only cohort. This would not have been so problematic had BGP-15 not induced further inhibition of protein synthesis or expression of the cytoskeletal mechanotransduction proteins, dystrophin and β-dystroglycan on top of that already elicited by IRI. That being said, we postulate that these effects of BGP-15 are specific to its activity in an IRI-induced cellular stress environment since induction of HSP-72 and repression of PARP-1 have been previously associated with enhanced protein synthesis and skeletal muscle accretion [97].

## 4. Materials and Methods

### 4.1. Animals

#### 4.1.1. Ethical Approval

All experimental procedures were approved by the Victoria University Animal Ethics Committee (AEETH15/006) and conformed to the Australian Code of Practice for the Care and Use of Animals for Scientific Purposes.

#### 4.1.2. Experimental Design and Treatments

Male Balb/c mice were acquired from the Animal Resource Centre (ARC, Western Australia) and randomly allocated to treatment groups (*n* = 8) upon arrival. Mice were housed on a 12-h light/dark cycle with ad libitum access to food (i.e., standard mice chow) and water supply throughout the experiments. Mice were administered either vehicle (VEH; 0.1% dimethyl sulfoxide (DMSO) in sterile water), irinotecan (IRI; 30 mg/kg (Sigma Aldrich, Sydney, Australia) dissolved in 0.1% DMSO) or BGP-15 adjuvant therapy with IRI (IRI+BGP; 15 mg/kg (BGP-15 donated by N-gene Research Laboratories, New York, NY, USA) dissolved in 0.1% DMSO). Treatments were administered via intraperitoneal injection six times over a fifteen-day period (i.e., on days 1, 3 5, 8, 10 and 12). The cumulative dose of IRI administered is equivalent to the 180 mg/m^2^ cumulative dose utilized in standard clinical regimens [98], as previously described by us [99], while the dose of BGP-15 is equivalent to that previously used by our group to protect against chemotherapy-induced skeletal myopathy [36]. Animals were weighed prior to the commencement of treatment (PRE) on each day of treatment and at the experimental endpoint. Food and water consumption were monitored throughout the duration of the treatment protocol.

### 4.2. Body Composition

Echo magnetic resonance imaging (echoMRI) was utilized to assess the effect of IRI treatment and BGP-15 adjuvant therapy on body composition indices of lean and fat mass. Live mice were placed into an echoMRI body composition analyzer (EMR-150, Echo Medical Systems, Houston, TX, USA) on day 1 (Pre) and day 15 (Post) of the treatment protocol, as previously described [8]. Lean and fat mass was quantified via triplicate scans spaced 30 s apart and reported as the mean of these triplicate scans.

### 4.3. Physical Activity Assessment

To evaluate the impact of IRI treatment and BGP-15 adjuvant therapy on the physical activity of mice, animals were individually housed for 24 h immediately prior to treatment commencement and the experimental endpoint in Promethion Metabolic cages (Sable Systems, Las Vegas, NV, USA). Cages allowed free access to food, water and a running wheel. Real-time voluntary activity associated with wheel running and general cage-based ambulatory activity was evaluated as described by us previously [8,36]. Data presented pertain to the total 24-h post-treatment period.

### 4.4. Surgery

At the conclusion of the treatment regimen and following the final echoMRI scan, mice were deeply anesthetized via isoflurane inhalation and administered an intraperitoneal injection of 0.040 μmol/g puromycin (Millipore, Sydney, Australia) dissolved in 100 μL of 0.9% saline to measure protein synthesis [45]. Thereafter, non-recovery surgery was performed. Muscles of interest were surgically excised for ex vivo analysis in the following order: (1) left and right flexor digitorum brevis (FDB) muscles for the measurement of mitochondrial functional parameters; (2) right extensor digitorum longus (EDL) and soleus (SOL) muscles for the assessment of contractile properties. Following this, at exactly 30 min after the injection of puromycin: (3) right tibialis anterior (TA) muscles were harvested and immediately snap-frozen for Western blotting experiments; and (4) the left TA was weighed and prepared for histological assessment prior to snap-freezing. The remaining tissues were then harvested (i.e., the left EDL and SOL and the heart), weighed and snap-frozen.

### 4.5. Ex Vivo Skeletal Muscle Contractile Function

Ex vivo evaluation of skeletal muscle contractile properties was performed as previously described by us [100,101,102], using the predominantly fast-twitch fiber EDL and the predominately slow-twitch fiber SOL muscles. These muscles were exposed and loops were tied at both tendons with 4.0 surgical silk thread before being dissected from the hindlimb and placed into individual organ baths of a Myodynamics Muscle Strip Myograph System (DMT-Asia Pacific, Bella Vista, Australia). Each organ bath was filled with Krebs solution (NaCl 118 mM, MgSO_4_·7H_2_O 1 mM, KCl 4.75 mM, Na_2_HPO 1 mM, CaCl_2_ 2.5 mM, NaHCO_3_ 24 mM and glucose 11 mM; pH 7.4) infused with carbogen (5% CO_2_ in O_2_) and maintained at a temperature of 30 °C. The proximal end of the muscle was tied to the force transducer, while the distal end was tied to a micromanipulator, with stimulating electrodes flanking the muscle belly. Data were collected and analyzed using LabChart Pro version 8.0 software (ADInstruments, Bella Vista, Australia). Initially, the optimal length (L_o_) of each muscle was established through the delivery of sequential twitch contractions, whilst incrementally stretching each muscle, until the point where the greatest force that was produced by the muscle was found and the length of the muscle at this point was measured with calipers. Once L_o_ was determined, a force–frequency protocol was performed utilizing supramaximal square wave 0.2-ms pulses at an incremental range of frequencies (i.e., 10, 20, 30, 40, 50, 60 80, 100, 120 and 150 Hz), with a 3-min rest period in between each stimulation to prevent fatigue. The training duration of pulses was 350 and 500 ms for the EDL and SOL muscles, respectively, due to their differing muscle fiber characteristics. The muscles were then stimulated with three single-twitch contractions for the analysis of basic contractile properties, i.e., peak twitch force (Pt), time to peak (TTP), half relaxation time (½ RT) and maximum rate of force development (df/dt). To investigate fatigue susceptibility, muscles next underwent a three-minute fatigue protocol, where EDL muscles were tetanically stimulated as above at 100 Hz, while SOL muscles were tetanically stimulated every two seconds at 80 Hz to elicit a comparable level of fatigue. Absolute force (P_o_) was considered as the force produced from the first tetanic response stimulated during the fatigue protocol. Specific force (sP_o_) was assessed as a normalized force relative to the physiological cross-sectional area (CSA) which takes into consideration three key variables of force production (i.e., muscle mass, optimal length and muscle density (∼1.06 mg mm^−3^)), and was calculated using the equation sP_o_ = P_o_ × (muscle mass/1.06 × L_o_ × fiber length:muscle length ratio), as previously described [103].

### 4.6. Mitochondrial Respiratory Phenotyping

#### 4.6.1. Flexor Digitorum Brevis Muscle Fiber Isolation

In order to conduct mitochondrial metabolic profiling, FDB muscles were harvested from both feet and fibers were isolated using a protocol established by Schuh et al. [104], as performed by us previously [36,102,105]. FDB muscles were incubated in 1 mL of pre-warmed dissociation medium (Dulbecco’s modified Eagle medium (DMEM), Gibco 10566016; 2% FBS, Bovogen Biologicals; 4 mg/mL collagenase A, Roche 10103586001; 50 µg/mL gentamycin, Sigma Aldrich, Sydney, Australia, G1397) for 1 h and 45 min (37 °C, 5% CO_2_). Following this dissociation period, FDB bundles were placed into ~1.5 mL of incubation medium (DMEM, high glucose, GlutaMAX™ supplement, Gibco/Thermo Fisher Scientific, Scoresby, Australia 10566016; 2% FBS, Bovogen Biologicals; 50 µg/mL gentamycin, Sigma Aldrich G1397) and triturated with pipette tips of decreasing bore size to yield single fibers.

#### 4.6.2. Seahorse Microplate Preparation

To facilitate fiber adherence to Seahorse XF24 cell culture V7 microplates (Seahorse Bioscience, Mulgrave., Australia), wells were coated with 5 µL of extracellular matrix (Sigma Aldrich, Sydney, AustraliaE1270) diluted in DMEM (1:1). Then, 75 µL of isolated FDB fibers was aliquoted into coated wells and confluency was determined using light microscopy. A minimum of ~60% confluency, i.e., the estimated percentage of the well bottom covered by isolated FDB fibers, was deemed optimal. If a well did not meet this requirement, an additional 50 µL aliquot of fibers was dispensed into wells.

#### 4.6.3. Mitochondrial Metabolic Profiling Using the XF24 Extracellular Flux Analyzer

Mitochondrial metabolic profiling was performed using extracellular flux analysis (Seahorse Bioscience/Agilent, Mulgrave, Australia) as described by us previously [105]. Basal, phosphorylating, maximal and non-mitochondrial respiration was measured initially through the sequential addition of 2 µg/mL oligomycin, 400 nm carbonyl cyanide-p-trifluoromethoxyphenylhydrazone (FCCP) with 10 mM sodium pyruvate, and 1 µM antimycin A, respectively. The extracellular acidification rate (ECAR) was concomitantly measured as an indicator of anaerobic glycolysis.

### 4.7. Skeletal Muscle Histology

All histological experiments were completed as previously described by us [36,105]. To determine whether IRI had atrophic effects on skeletal muscle and, subsequently, whether BGP-15 adjuvant therapy could rescue any atrophy, we histologically assessed TA muscles which were cryopreserved in optimal cutting temperature compound (Sakura Finetek, Maumee, OH, USA). TAs were sectioned (10 µm, −20°C, Leica CM1950, Leica Biosystems, Mount Waverley, Australia) and mounted onto glass slides and then stained with hematoxylin and eosin (H&E) to evaluate muscle fiber size through CSA. All slides were imaged on a Zeiss Axio Imager Z2 microscope (Carl Zeiss MicroImaging GmbH, Oberkochen, Germany) at 20× magnification and analyzed as described previously [36,105] using ImageJ software (NIH, Bethesda, MD, USA).

### 4.8. Western Blot Analyses

Western blotting was utilized to explore the effect of IRI treatment and BGP-15 adjuvant therapy on molecular signaling pathways surrounding protein synthesis and degradation, cell stress and membrane structure. All Western blotting protocols were completed as previously described by us [45,105]. Frozen TA muscles were homogenized using an Omni Tissue Homogenizer (TH220, Omni International, Kennesaw, GA, USA) for 20 s in ice-cold Western immunoprecipitation kinase (WIK) buffer (40 mM Tris, pH 7.5; 1 mM ethylenediaminetetraacetic acid (EDTA); 5 mM etheylen glycol-bis(β-aminoethyl ether)-N, N, N’, N’-tetraacetic acid (EGTA); 0.5% TritonX-100; 25 mM β-glycerophosphate; 25 mM NaF; 1 mM Na3VO4; 10 g/mL leupeptin; and 1 mM phenylmethylsulfonyl fluoride (PMSF). Muscle homogenate was centrifuged at 3500 rpm for 5 min at 4 °C, before the pellet was resuspended and the muscle homogenate was frozen for further analysis. Protein concentrations were determined using a sample assay kit (Bio-Rad Laboratories, Hercules, CA, USA) to ensure equal loading on the gels. Samples were prepared with equivalent amounts of protein in either 2× sodium dodecyl sulfate (SDS) sample buffer (20% (v/v) glycerol; 100 mM Tris-Base, pH 6.8; 4% (w/v) SDS; 0.017% (w/v) bromophenol blue; 0.25 M dithiothreitol (DTT)) or 4× Laemmli buffer (Bio-Rad Laboratories, Hercules, CA, USA) reduced in 50 mM DTT, heated for 5 min at 95 °C and subjected to electrophoretic separation on 7.5–12% SDS-acrylamide gels. Antibodies that required exceptions to this protocol include Total oxidative phosphorylation (OXPHOS) cocktail (1:1000; ab110413; Abcam, Cambridge, UK), where samples were heated for 5 min at 40 °C, and anti-α-sarcoglycan (1:200; IVD3(1)A9; Developmental Studies Hybridoma Bank (DSHB)), which was probed in samples under non-denaturing and non-reducing conditions, as per supplier recommendations. Following electrophoretic separation, proteins were transferred to a polyvinylidene fluoride membrane, blocked with 5% not-fat milk powder in Tris-buffered saline containing 0.1% Tween 20 (TBST) for 1 h followed by an overnight incubation at 4 °C with primary antibody dissolved in TBST containing either 1% BSA or 3% non-fat milk powder. The following primary antibodies were used: anti-phospho 4E-BP1^(Thr37/46)^ (1:1000; #2855; Cell Signalling Technology (CST, Danvers, MA, USA)), anti-4E-BP1 (1:1000; #9452; CST), anti-4-HNE (1:1000; ab46545; Abcam, Cambridge, UK), anti-phospho Akt^Ser473^ (1:2000; #4060; CST), anti-phospho Akt^Thr308^ (1:2000; #13038; CST), anti-Akt (1:1000; #4691; CST), anti-Atrogin-1 (1:1000; AP2041; ECM Biosciences, Versailles, KY, USA), anti-β-Dystroglycan (1:500; MANDAG2 (7D11); DSHB), anti-Cytochrome C (1:2000; #11940, CST), anti-δ-Sarcoglycan (1:1000; ab137101; Abcam), anti-Desmin (1:1000; #5332; CST), anti-DJ-1 (1:1000; #5933; 1:1000), anti-Dystrophin (1:200; ab15277; Abcam), anti-phospho eIF2a^Ser51^ (1:1000; #3398; CST), anti-eIF2a (1:1000; #5324; CST), anti-phospho ERK1/2^(Thr202/Tyr204)^ (1:1000; #9101; CST), anti-ERK1/2 (1:1000; #9102; CST), anti-HO-1 (1:1000; ADI-SPA-896; Enzo Life Sciences, Farmigdale, NY, USA), anti-HSP-70 (1:1000; ADI-SPA-812; Enzo Life Sciences), anti-phospho JNK^(Thr183/Tyr185)^ (1:1000; #4668; CST), anti-JNK (1:1000; #9252; CST), anti-Keap-1 (1:1000; #8047; CST), anti-Laminin (1:2000; L9393; Sigma-Aldrich, Sydney, Australia), anti-MMP-2 (1:1000; ab92536; Abcam), anti-MMP-9 (1:300; AF909; R&D Systems), anti-MuRF-1 (1:200; AF5366; R&D Systems, Minneapolis, MN, USA); anti-NRF-2 (1:1000; #12721, CST), anti-phospho p38^(Thr180/Tyr182)^ (1:1000; #4511; CST), anti-p38 (1:1000; #9212; CST), anti-phospho p70s6k^(Thr389)^ (1:1000; #9234; CST), anti-p70s6k (1:1000; #2708; CST), anti-PARP-1 (1:1000; 9542, CST), anti-Puromycin (1:5000; MABE343; Merck Millipore, Bayswater, Australia), anti-Rac1 (1:500; #05-389; Merck Millipore), anti-SOD1 (1:3000; ADI-SOD-101; Enzo Life Sciences) and anti-Ubiquitin (1:3000; sc8017; Santa Cruz, Dalla, TX, USA). After overnight incubation, membranes were washed 3 separate times for 10 min each in TBST and then probed with a horseradish peroxidase-conjugated secondary antibody (1:5,000; anti-rabbit IgG, 1:5,000; anti-goat IgG or 1:20,000; anti-mouse IgG, Vector Laboratories, Burlingame, CA, USA) in 5% not-fat milk powder in TBST for 1 h at room temperature. Membranes that were probed with anti-Puromycin were incubated in anti-mouse IgG Fc 2a-specific horseradish peroxidase-conjugated secondary antibody (1:50,000; Jackson ImmunoResearch Laboratories Inc., West Grove, PA, USA), as previously described [45]. Following another set of 3 separate 10-min washes in TBST, the blots were developed with a DARQ CCD camera mounted to a Fusion FX imaging system (Vilber Lourmat, Eberhardzell, Germany) using ECL Prime reagent (Amersham, Piscataway, NJ, USA). Once images were captured, the membranes were stained with Coomassie Blue and then normalized to total protein. Densitometric measurements were carried out using FusionCAPTAdvance software (Vilber Lourmat, Eberhardzell, Germany).

### 4.9. Citrate Synthase Activity

Citrate Synthase (CS) activity was measured as a marker of mitochondrial density and/or anaplerosis. Homogenized TA muscles in WIK buffer (as described above) were added to the reagent cocktail (100 mM Tris Buffer, 1 mM 5,5′-Dithiobis(2-nitrobenzoic acid) (DTNB) and 3 mM Acetyl CoA), and to initiate the reaction, oxaloacetate (10 mM) was added just prior to measuring CS activity spectrophotometrically (412 nm, 25 °C, 5 min). CS activity was calculated using the extinction coefficient of 13.6 [106] and expressed relative to muscle wet weight.

### 4.10. Zymography Analyses for Gelatinase Activity

Zymography was performed via gelatin-infused SDS-PAGE to detect the gelatinolytic activity of matrix metalloproteinases (MMPs), specifically the isoforms MMP-2 and MMP-9, based on a previously established protocol [56]. EDL muscles were placed in ice-cold zymography homogenization buffer (50 mM Tris-HCl; 150 mM NaCl; 10 mM CaCl_2_; pH 7.5) and were homogenized using the Omni Tissue Homogenizer (TH220, Omni International, Kennesaw, GA, USA) for 3 sets at medium speed for 8 s. Muscle homogenate was centrifuged at 3500 rpm for 5 min at 4 °C, before the pellet was resuspended and the muscle homogenate frozen for further analysis. Protein concentrations were determined using an RC DC sample assay kit (Bio-Rad Laboratories, Hercules, CA, USA) to ensure equal loading on the gels. Samples were then prepared in 4X SDS sample buffer (400 mM Tris-HCl, pH 6.8; 4% (w/v) SDS; 20% glycerol (v/v); 0.005% (w/v) bromophenol blue) under non-reducing, non-denaturing conditions, with 100 µg of protein loaded onto 0.75-mm thick 7.5% SDS-acrylamide gels that were co-polymerized with 1% (w/v) gelatin (Sigma, Sydney, Australia). After gel electrophoresis was completed, gels were washed twice for 40 min in denaturing buffer (2.5% (v/v) Triton-X; 50 mM Tris-HCl; 5 mM CaCl_2_; pH 7.6) to remove SDS. After this, gels were washed twice for 15 min in renaturing buffer (50 mM Tris-HCl; 5 mM CaCl_2_; pH 7.6) before being incubated overnight (~18 h) at 37 °C in developing buffer (50 mM Tris-HCl; 150 mM NaCl; 10 mM CaCl_2_; 1 µM ZnCL_2_; 0.02% (w/v) NaN_3_; pH 7.5). The gels were then stained for 3 h in Coomassie Blue solution (0.05% (w/v) Coomassie Brilliant Blue; 30% (v/v) methanol; 10% (v/v) acetic acid) before being incubated for 3 h in de-stain solution (30% (v/v) methanol; 10% (v/v) acetic acid), allowing the visualization of the digestion of gelatin (i.e., gelatinase activity) against the dark blue background. Gels were imaged using an Epson Perfection V700 Photo Scanner (Epson, Sydney, Australia). Samples were run in duplicate on separate gels, with images semi-quantitatively analyzed using Image J software (NIH, Bethesda, MD, USA), according to a previously established method [107], with the mean area density reported.

### 4.11. Statistics

Data are presented as mean ± standard error of the mean (SEM). Data were analyzed using GraphPad prism v8 (GraphPad Software, San Diego, CA, USA). A one-way ANOVA with Tukey’s post-hoc test was utilized to detect treatment differences for parametric data, while Dunn’s multiple comparison test was utilized to analyze non-parametric data, with an α-value of 0.05 considered significant. A two-way repeated measures ANOVA was used to detect differences between treatment and stimulation frequency/time for force–frequency relationships and fatigue susceptibility studies, with Tukey’s post-hoc test utilized for multiple comparisons.

## 5. Conclusions

This study shows, for the first time, that, independent of cancer, IRI treatment diminishes body mass indices, underlined by reduced skeletal muscle mass and, subsequently, contractile function, which were mitigated by the PARP inhibitor BGP-15. The protective effect of BGP-15 adjuvant therapy occurred alongside a paradoxical reduction in protein synthesis rates, likely underlined by aberrant proteostasis; however, this needs to be confirmed. Additionally, IRI treatment and BGP-15 reduced mTORC2 signaling and caused changes to the expression of cytoskeletal mechanotransduction proteins associated with the DAPC in a step-like manner that mirrored the reduction in protein synthesis rates. These data highlight remodeling of the contractile apparatus, cytoskeleton and/or ECM that is induced by IRI and even more so by IRI+BGP-15. It is difficult to conclude from our data whether BGP-15, when teamed with IRI treatment, is beneficial for the skeletal muscular system or not, since on the one hand, BGP-15 mitigated many of the cachectic side-effects of IRI treatment, but on the other hand, it made fast-twitch muscles more prone to tearing, despite rescuing function. Time-course studies should be undertaken to determine whether the down regulation of DAPC constituent proteins evoked by both IRI and IRI+BGP-15 induces a muscular dystrophy-like phenotype in the long term. This aspect should be of significant clinical consideration before instigating the mainstream use of PARP inhibitors in anti-cancer treatment and when prescribing IRI-based chemotherapy regimens to patients, since the maintenance of body and, particularly, skeletal muscle mass improves patient survival and long-term outcomes during cancer treatment.

## Supplementary Materials

The following are available online at https://www.mdpi.com/2072-6694/12/12/3810/s1, Figure S1: Full-length Western blot images relating to whole membrane related proteins, Figure S2: Full-length Western blot images relating to the data presented in Figure 3 and Figure 4, Figure S3: Full-length Western blot images relating to the data presented in Figure 6 and Figure 7, Figure S4: Protein expression of antibodies that were probed for levels of phosphorylated and total protein, Table S1: Densitometry summary data from representative Western blot images.
