# Supplementary material for: The Paradoxical Effect of PARP Inhibitor BGP-15 on Irinotecan-Induced Cachexia and Skeletal Muscle Dysfunction
Source: Cancers (Basel). 2020 Dec 17;12(12):3810. doi: 10.3390/cancers12123810 (PMC7766767; doi:10.3390/cancers12123810)
Supplement: Supplementary file 1 [file cancers-12-03810-s001.pdf]

Supplementary Materials:

The Paradoxical Effect of PARP Inhibitor BGP-15 on Irinotecan-Induced Cachexia and Skeletal Muscle Dysfunction

Dean G. Campelj, Cara A. Timpani, Aaron C. Petersen, Alan Hayes, Craig A. Goodman and Emma Rybalka

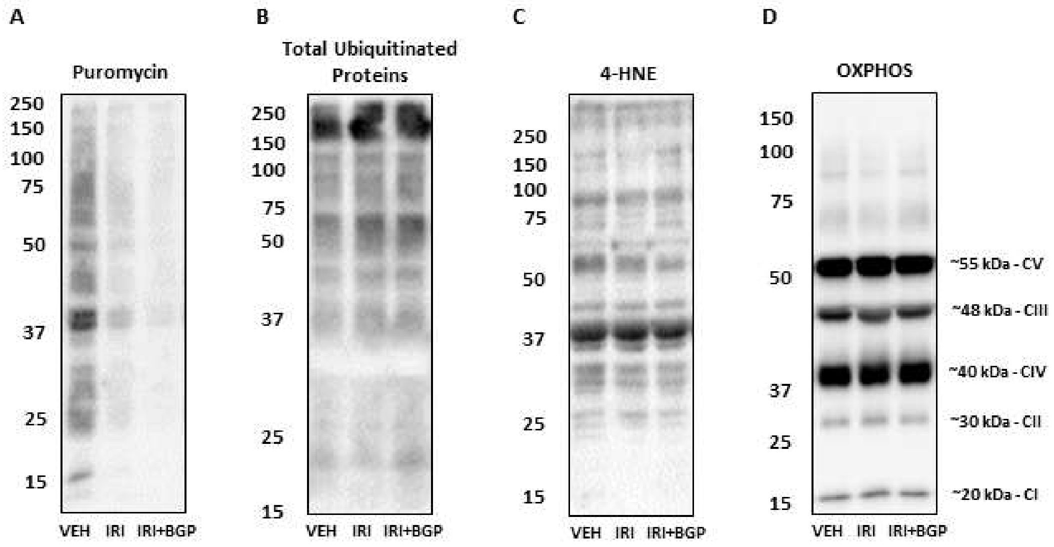

Figure S1. Full-length Western blot images relating to whole membrane related proteins. The above images display the largest available vertical membrane area probed with each respective primary antibody that requires the whole full area of the membrane for analysis.

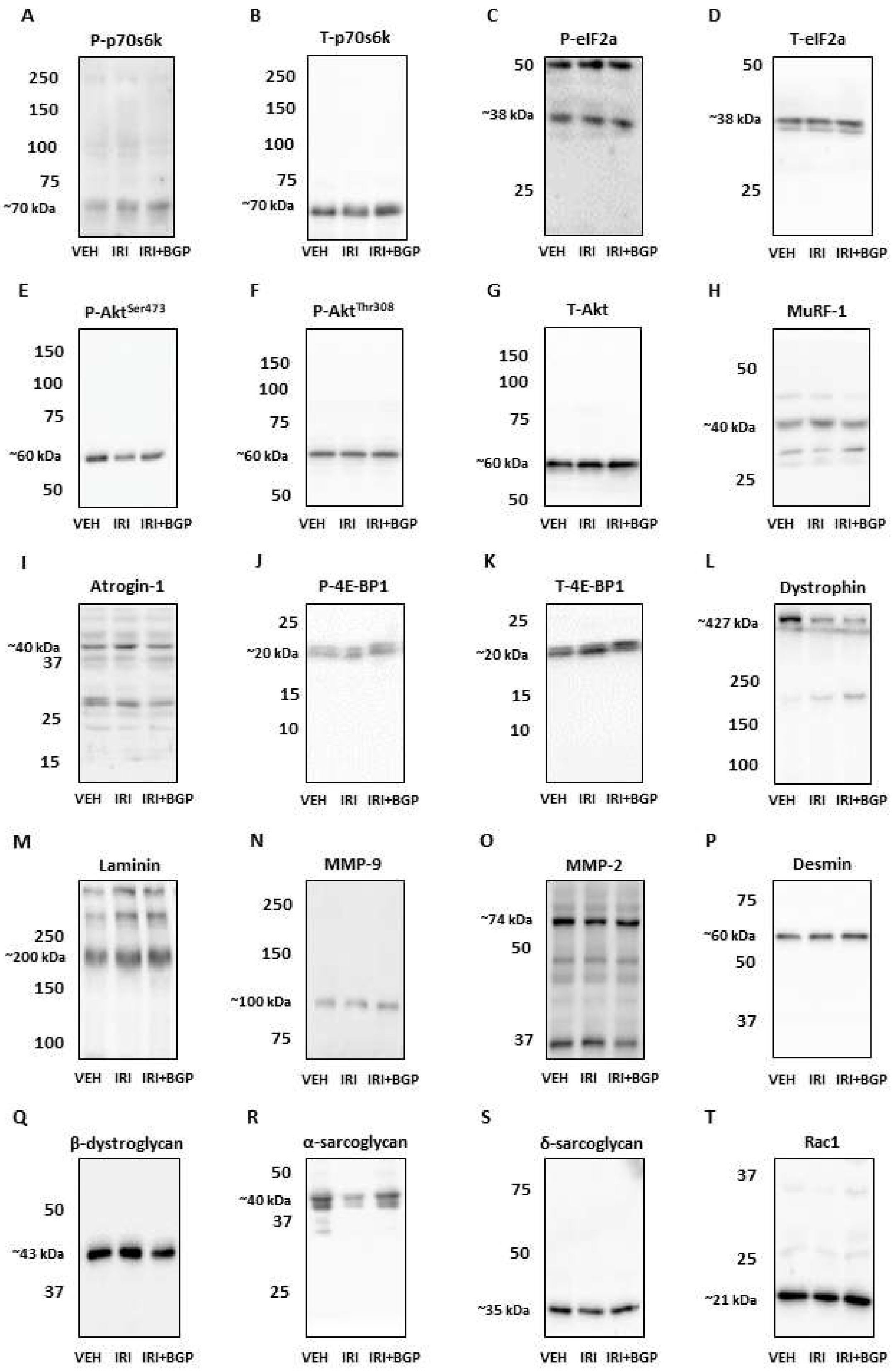

Figure S2. Full-length Western blot images relating to the data presented in Figure 3 and 4. After transferring the protein from the gel to the PVDF membrane, membranes were cut horizontally to allow for probing with multiple primary antibodies on a single membrane/gel. Subsequently, the

above images display the largest available vertical membrane area probed with each respective primary antibody.

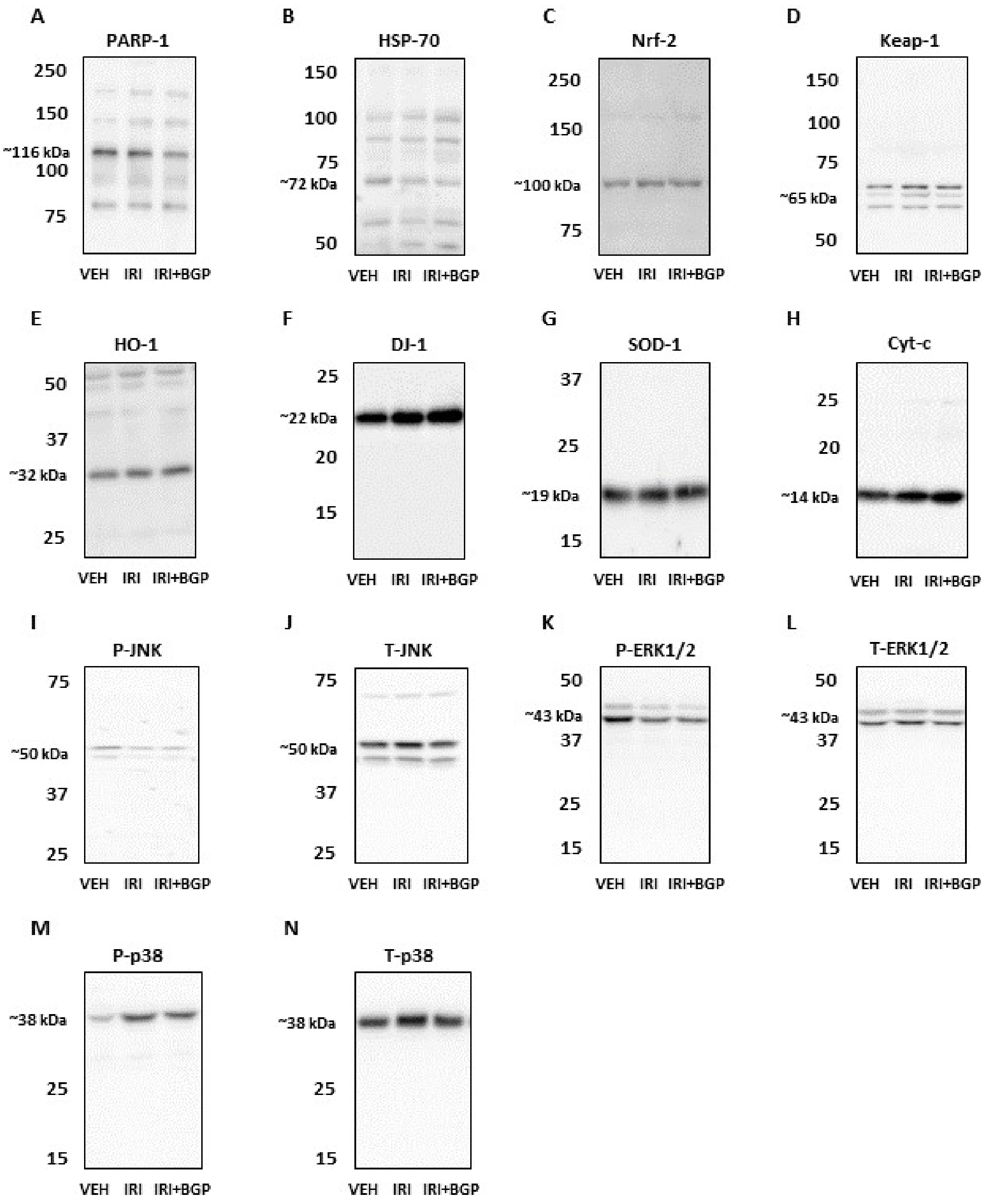

Figure S3. Full-length Western blot images relating to the data presented in Figure 6 and 7. After transferring the protein from the gel to the PVDF membrane, membranes were cut horizontally to allow for probing with multiple primary antibodies on a single membrane/gel. Subsequently, the above images display the largest available vertical membrane area probed with each respective primary antibody.

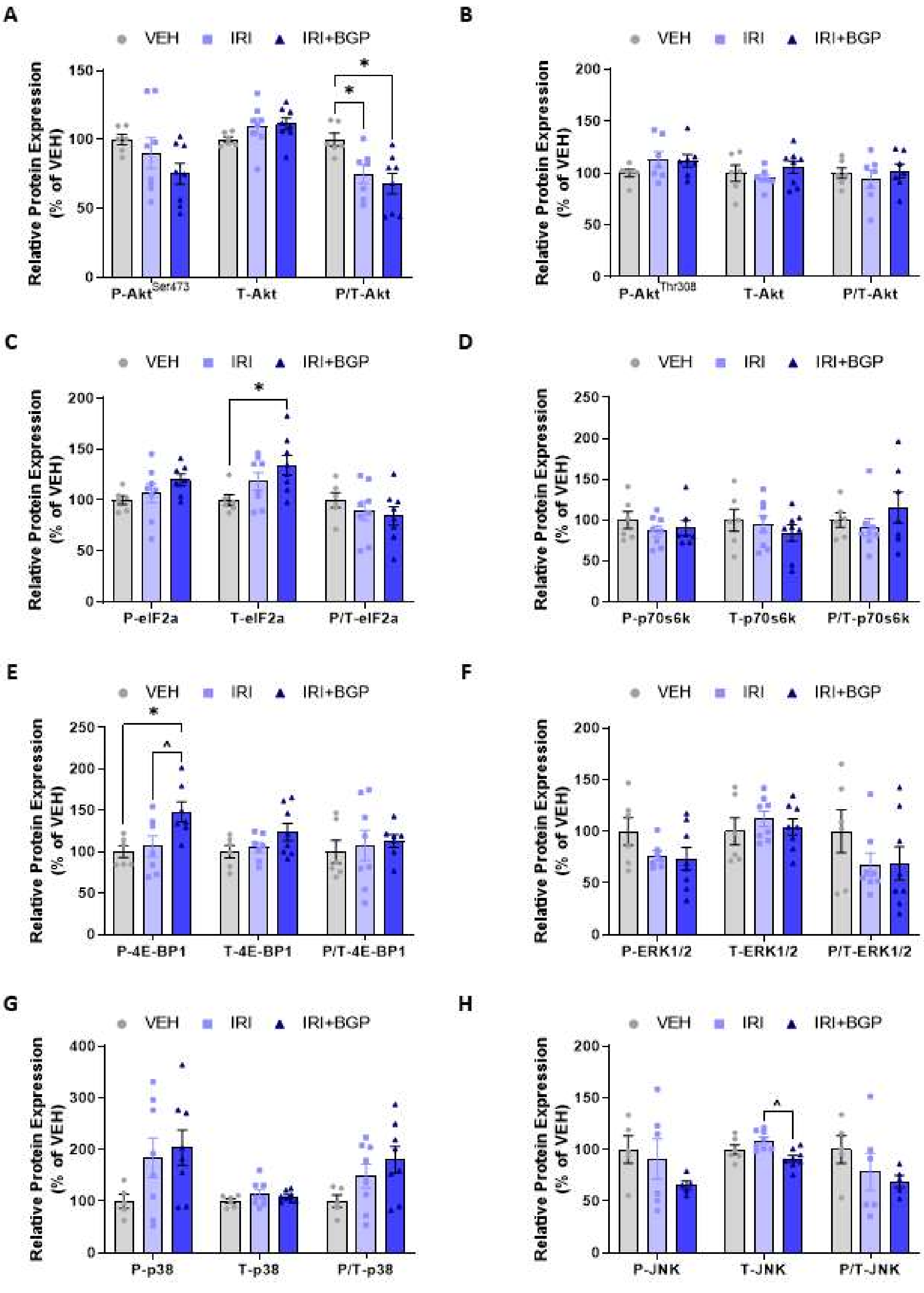

Figure S4. Protein expression of antibodies that were probed for levels of phosphorylated and total protein. Data is presented as phosphorylated, total and phosphorylated to total protein ratios for all relevant antibodies analyzed from Figures 3 and 7. * = $p < 0.05$ compared to VEH, ^ = $p < .05$ compared to IRI; n = 5-8.

Table S1. Densitometry summary data from representative Western blot images. Data is presented as integrated density relative to total protein (in arbitrary units, i.e. (a.u.)) for all antibodies analysed.

	Protein/Total Protein (a.u.)				Protein/Total Protein (a.u.)		
**Target Protein**	**VEH**	**IRI**	**IRI+BGP**	**Target Protein**	**VEH**	**IRI**	**IRI+BGP**
P-4E-BP1	23.42	18.84	28.04	T-JNK	101.62	131.27	95.74
T-4E-BP1	98.65	128.02	114.52	Keap-1	26.71	34.59	29.73
4-HNE	490.50	415.20	405.86	Laminin	227.35	332.57	321.20
P-Akt^{Ser473}	75.03	42.30	57.88	MMP-2	246.10	253.64	272.18
P-Akt^{Thr308}	10.42	11.33	10.90	MMP-9	7.57	5.22	6.63
T-Akt	51.08	56.00	68.76	MuRF-1	34.70	46.79	38.20
α-Sarcoglycan	38.78	10.20	32.27	NRF-2	222.90	238.51	284.66
Atrogin-1	36.75	50.41	34.45	OXPHOS - CV	158.61	172.78	154.48
β-Dystroglycan	40.13	44.37	33.46	OXPHOS - CIV	94.52	91.39	96.14
Cytochrome C	84.85	111.62	119.14	OXPHOS - CIII	49.85	46.69	44.55
δ-Sarcoglycan	71.02	62.05	66.03	OXPHOS - CII	11.48	13.09	10.79
Desmin	188.93	102.59	114.61	OXPHOS - CI	13.05	13.95	12.19
DJ-1	76.89	84.92	105.31	P-p38	150.83	474.87	383.84
Dystrophin	18.82	8.01	6.46	T-p38	675.91	831.08	693.80
P-eIF2a	21.57	20.28	18.42	P-p70s6k	10.60	8.56	10.69
T-eIF2a	43.74	44.37	48.23	T-p70s6k	37.10	36.01	40.91
P-ERK1/2	437.48	273.87	244.08	PARP-1	68.13	62.24	40.74
T-ERK1/2	254.34	344.56	288.11	Puromycin	34.42	11.70	7.97
HO-1	80.51	80.26	91.81	Rac1	201.62	180.40	218.92
HSP72	7.93	8.24	11.42	SOD1	463.32	530.60	560.12
P-JNK	16.52	7.30	8.69	Ubiquitin	32.07	36.01	25.57
